# Multiplexed catheter-integrated pressure sensing system for endoluminal interventions

**DOI:** 10.1038/s41378-026-01411-0

**Published:** 2026-09-15

**Authors:** Xiaotong Guo, Qindong Zheng, Jinshi Zhao, Bing Li, Eric M. Yeatman

**Affiliations:** 1 Department of Electrical and Electronic Engineering, Imperial College London, London, UK; 2 Department of Bioengineering, Imperial College London, London, UK; 3 Department of Metabolism, Digestion, and Reproduction, Imperial College London, London, UK; 4 Institute for Materials Discovery, University College London, London, UK; 5 College of Science and Engineering, University of Glasgow, Glasgow, UK

**Keywords:** Engineering, Materials science

## Abstract

Advances in flexible catheters pave the way for minimally invasive diagnosis and treatment of luminal organs and tubular structures through endoluminal interventions. A key challenge is in establishing non-constraining pressure monitoring at the interfaces between medical catheters and intraluminal anatomy, where catheter–tissue interactions may be influenced by lumen curvature, structural variability, and physiological motion. In this work, we present a scalable and multi-purpose pressure sensing system for multidirectional monitoring of tissue interactions, establishing a robust solution for deploying diagnostic and therapeutic instruments in various types of endoluminal interventions. This approach provides an integrated pressure sensing platform that combines a thin-film piezoelectric sensor array with a bespoke multi-lumen catheter and a custom signal acquisition circuit. The sensor array is fabricated from an ultrathin poly (vinylidene fluoride-co-trifluoroethylene) (P(VDF-TrFE)) film using a multilayer sandwich architecture with patterned gold electrodes, resulting in a flexible device with a total thickness of approximately 20 µm and sensing units as small as 2.25 mm^2^. The multi-lumen catheter is fabricated with a cost-effective and highly scalable fiber drawing technology, establishing a means of fast prototyping catheters with bespoke microstructures for sensor integration and medical instrument deployment. Supported by a custom high-impedance acquisition circuit, the system achieves a sensitivity of 16 mV per kPa, which is approximately 25 times higher than state-of-the-art catheter-integrated sensors, while maintaining a sensing range from 0 to 80 kPa. Through in-vitro phantom studies, the system performs precise multi-directional sensing within various clinical endoluminal scenarios, showing its potential in digitalizing tissue interactions during endoluminal interventions.

## Introduction

Flexible instruments have gained increasing popularity in many minimally invasive endoluminal interventions due to their advantages in navigating through tortuous passage^1,2^. The catheter-based intervention represents one of the most promising flexible instrument applications for diagnostic and therapeutic tools implementation covering endovascular (e.g., stent deployment), gastrointestinal (e.g., colonoscopy), and bronchial (e.g., bronchoscopy) interventions^3,4^. Manipulation of catheters during surgeries is normally assisted by image-guided technology. However, such manipulation shows an absence of intrabody monitoring of subtle pressure variations in the vicinity of catheter contact with surrounding intralumenal structures. As a result, catheter interventions suffer from suboptimal interactions with surrounding tissues, reduced coupling efficiency, and unawareness of sudden increase in pressure^5^. Such drawbacks lead to a heightened risk of tissue damage and longer recovery time^6^, and limited capability to detect small, unnatural protrusions within the complex natural of intraluminal anatomy, such as aneurysm^7^.

Integrating catheters with sensitive, miniaturized and flexible pressure sensors can be a robust and novel approach to resolve these challenges^3,6,8^. Emerging strategies in the miniaturization of soft material pressure sensors, encompassing capacitive^9,10^, resistive^11,12^, and piezoelectric^13,14^ mechanisms, demonstrate considerable potential to advance progress in this context^15^. Recent advances in flexible sensor array have established versatile means in instrumenting balloon catheters for multiplexed tissue contact measurements on the balloon^4,16,17^. However, strategies for monitoring tissue interactions surrounding catheter bodies during navigation still remain limited. Many designs suffer from limited miniaturization and spatial resolution, leading to insufficient mapping for confined and complex intraluminal anatomy. Low sensitivity limits the measurement of subtle pressure, while high flexibility is needed for integration with millimeter-sized, tube-shaped catheters. Additionally, compatibility with commercial devices is another challenge, leading to difficulties in both electronic aspects (such as wiring connections) and mechanical aspects (such as sensor attachment).

Piezoelectric pressure sensor is a promising candidate to resolve the above challenges in sensitivity, flexibility, and miniaturization^18,19^. A high sensitivity is demonstrated in piezoelectric-based sensors due to their effectiveness in measuring subtle pressure changes while retaining a large measurement range^20,21^, making them naturally suitable for intrabody pressure measurement. Flexibility in piezoelectric materials can be retained by the recent applications of copolymers or derivatives, such as poly(vinylidene fluoride)-co-trifluoroethylene (P(VDF-TrFE))^22,23,24^. These novel piezoelectric materials exhibit lower elastic modulus than the conventional brittle substrates made of lead zirconate titanate^23,25^. Their integration with flexible substrates shows a simpler fabrication process and a higher conformity with soft geometry, making the piezoelectric-based sensors more suitable in integration with flexible instruments. Miniaturization of device made of piezoelectric materials can be achieved by sandwiching the active material between electrodes with methods such as spin-coating and evaporating deposition, which also presented as agile means in structing a web of sensing array^26,27^.

In line with the pressure sensor design, the compatibility of the pressure sensor with catheters is essential to its practical use in clinical settings. However, designs of commercial catheters are usually compact and self-contained, making less room for the sensor integration^3^. One solution is to fabricate a bespoke catheter with lumens for sensor coupling and accommodation for catheter-based instruments, as proposed in this paper. Thermal drawing is a low-cost and highly scalable technique for fabricating medical fiber with designated structure and versatile material. The process involves thermally softening an amorphous polymer-based, macro-structured fiber preform that has the same cross-sectional design as the target fiber^28^. The preform is then elongated into millimetre- or micrometre-scale fibers by applying an axial pulling pressure while maintaining the cross-sectional shape throughout the drawing process^29,30^. In recent decades, fibers produced using this technique have been studied for medical applications in sensing^31,32,33^, actuation^34,35^, robotics^36,37^, and smart wearable devices^2^.

Herein, we present a multiplexed, piezoelectric-based, and catheter-integrated pressure sensing system for real-time monitoring of catheter–tissue interactions during endoluminal interventions. The main contribution of this work is a system-level pressure feedback platform that combines a compact multiplexed P(VDF-TrFE)-based sensor array, a customized thermally drawn multi-lumen catheter, and a dedicated multi-channel signal acquisition module for real-time visualization. The sensor array is fabricated by sandwiching a thin P(VDF-TrFE) film between two patterned electrode layers, creating a compact and reconfigurable layout that can be integrated onto the curved catheter surface. The bespoke catheter is constructed with a central working lumen for medical instrument deployment and multiple side lumens for organized electrical routing, enabling the sensor layout and catheter structure to be co-designed as an integrated sensing interface. Coupled with the customized signal acquisition module, the system converts localized catheter–tissue contact into channel-dependent pressure signals during catheter manipulation. In this way, the proposed platform establishes a catheter-specific, multi-directional pressure feedback architecture for monitoring tissue interactions in confined endoluminal environments.

## Results

### Overview of catheter-integrated pressure sensing system

**Fig. 1:**
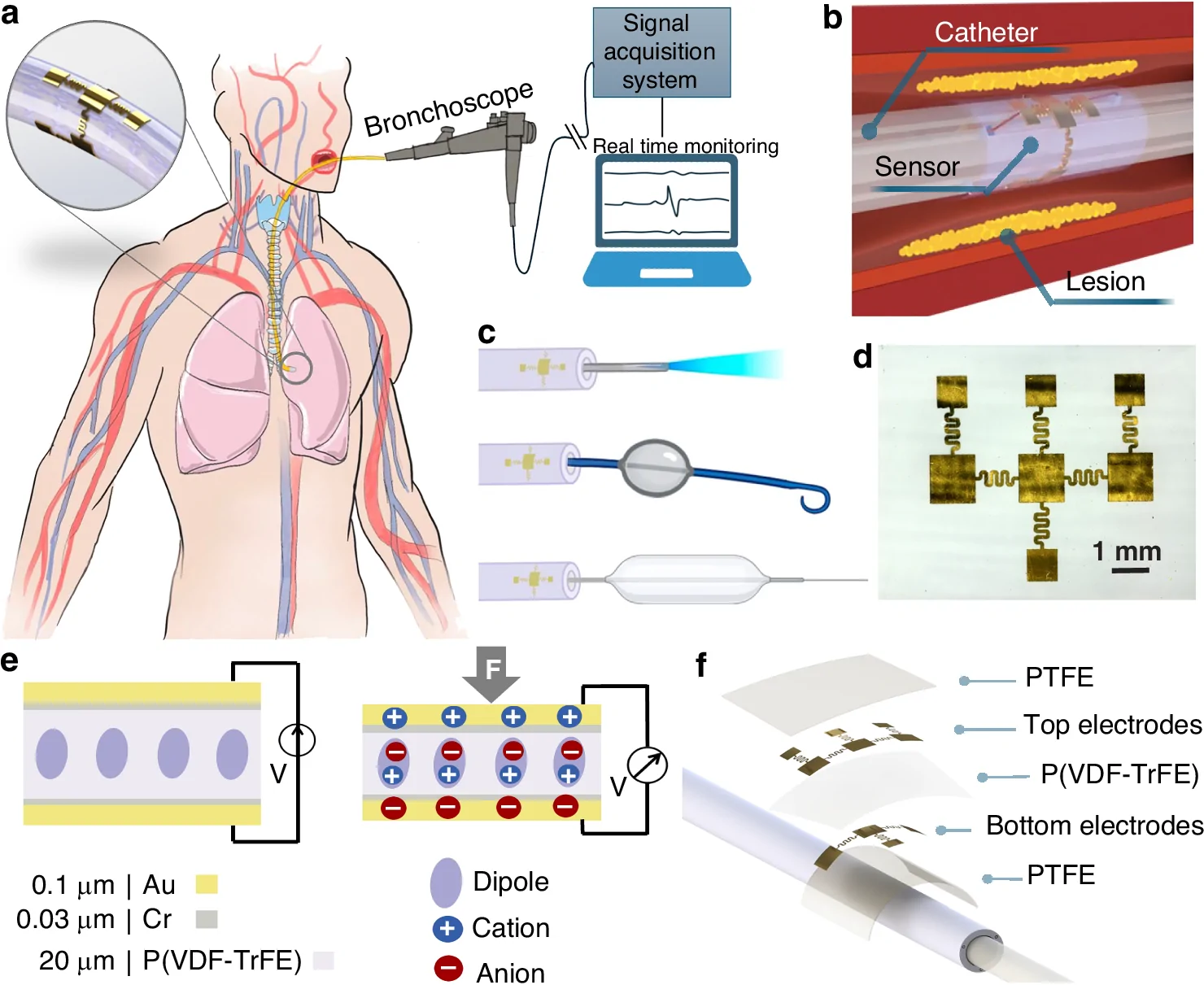
**Illustration of the catheter-integrated sensing system**. **a** Overview of the catheter-integrated pressure sensing system as applied in bronchial interventions. **b** Schematic of the catheter-integrated sensing system being in touch with an aneurysmal protrusion within a confined vascular structure. **c** Illustration of the catheter-integrated sensing system accommodating various medical devices, including optical catheter (top), urinary catheter (middle), and balloon catheter for stent deployment (bottom). **d** Microscopic view of the sensor array. **e** Cross-sectional schematics of the sensor design and piezoelectric mechanism showing the generation of potential difference when mechanical force is applied. **f** Demonstration of the device array in multilayer format integrated at the distal end of the catheter

The catheter-integrated pressure sensing system is designed in a miniaturized scale to navigate through confined intraluminal anatomy and provide real-time pressure feedback visualization (Fig. 1a,b). The integrated central working lumen of the catheter allows deployment of various diagnostic and therapeutic devices, such as optical catheter, balloon catheter, and urinary catheter (Fig. 1c). Through the adoption of a multilayer sandwich P(VDF-TrFE) thin-film fabrication technique, the sensor array is configured with a miniaturized size of 9 × 8 mm as illustrated in Fig. 1d. The sandwich layout provides an ultra-thin cross-sectional profile (around 20 µm) and enables signal readout when external mechanical loadings applied (Fig. 1e). The array is composed of three sensing elements (1.5 × 1.5 mm) wiring to their corresponding anode electrodes (1 × 1 mm), and a ground electrode (1 × 1 mm) is connected to all sensing elements. The electrodes are formed with one side of Au coating, while the sensing elements are formed by sandwiching the P(VDF-TrFE) thin film between two Au deposited layers. The wiring of all electrodes and sensing elements is built in the form of stretchable gold pattern (0.1 mm width) to provide a stretchability in axial and longitudinal directions.

Leveraging the flexibility and miniature design of P(VDF-TrFE) sensor, the fabricated sensor is conformed intimately to the curved geometry of medical catheters by wrapping over the distal end of the catheter body, as shown in Fig. 1f. This sensor-catheter integration design enables multi-directional contact pressure monitoring for the navigation within confined intraluminal spaces. Given the optimized design of the bespoke catheter, conductive wires are embedded inside the catheter to avoid exposure to intraluminal environment. After soldering electrodes and external wires, the sensor array is then sandwiched by two protective layers (Polytetrafluoroethylene (PTFE) with a thickness of 0.1 mm).

### Design and scalable fabrication of the multi-lumen catheter

To enable seamless sensor integration and multifunctionality for versatile therapeutic and diagnostic instruments, the bespoke multi-lumen catheter was designed with a central hollow working lumen surrounded by multiple side lumens arranged around its circumference. The catheter has an outer diameter of 2.8 mm and a central lumen of 2.2 mm, allowing the passage of standard medical instruments up to 2.0 mm in diameter, such as endoscopes and guidewires. The cross-sectional design was optimized to support sensor configuration, with equilateral distribution of side lumens (each 100 μm in diameter) that facilitates organized routing of wires (80 μm in diameter) to connect anode and ground electrodes without overlapping or crossing.

**Fig. 2:**
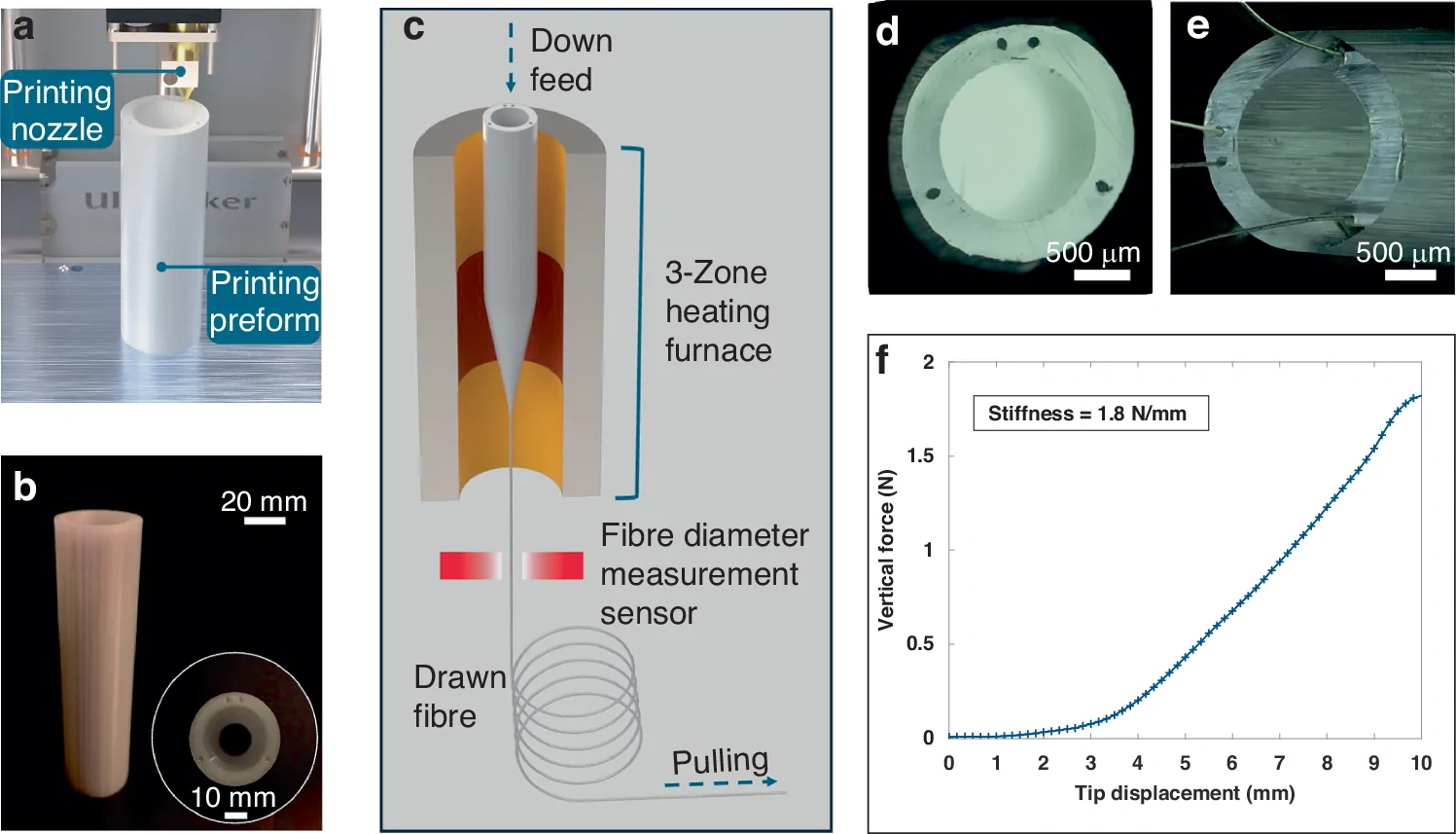
**Catheter fabrication and characterization**. **a** 3D printing of the preform with designated cross-section prepared for catheter fabrication. **b** Resulting 3D-print preform. **c** Schematic illustration of the fiber drawing process, where the colored hollowed cylinder represents the heating furnace. **d** Labeled microscopic cross-section view of the drawn fiber with channels 1-3 (the wiring channels for connecting anodes), 4 (the wiring channel for connecting cathode), and a central working channel for accommodating medical devices. **e** Microscopic view of the fiber with wires embedded. **f** Stiffness measurement of the fiber with a flexural rigidity test

For catheter fabrication, a scalable and low-cost fiber drawing process was used to produce polymeric fibers from three-dimensional (3D) printed preforms (Fig. 2a). This preform fabrication method has been proven as a robust approach that enables low cost, rapid production, and design flexibility, as reported in previous studies^36–39^. The preform material was selected as polycarbonate (PC) from various thermally drawable thermoplastic candidates (e.g., poly (methylmethacrylate) and acrylonitrile butadiene styrene) due to its good biocompatibility, high mechanical strength, and dimensional stability^36^. The printed PC preform configures a 39.2 mm diameter, a 14-fold ratio to catheter’s dimensions, and a length of 170 mm (Fig. 2b). A continuous fiber exceeding 3 meters in length was fabricated from a single preform, maintaining a diameter tolerance within ±0.2 mm with the fiber drawing technique (Fig. 2c). A 0.6-meter segment with consistent cross-sectional integrity was selected for use as the catheter body, providing sufficient length for endoluminal interventions.

Figure 2d shows the microscopic view of the cross-section of the resulting drawn fiber. The cross-section structure was well retained compared with the design of fiber geometry, allowing passing through the conductive wires therein (Fig. 2e). The layout of the side lumens facilitates streamlined wiring of the integrated sensor that has electrodes uniformly distributed at 120° intervals. Figure 2f illustrates the stiffness characterization results of the fabricated fiber based on a flexural rigidity test. The fiber with embedded wires shows a stiffness of 1.8 N/mm for an identical 2.8 mm diameter and 50 mm length, according to the equation ${Stiffness}={Force}/{Displacement}$.

Compared with other medical devices fabricated using mold-derived thermal drawing techniques, the use of the 3D-printed preform offers practical advantages in prototyping speed, lower fabrication cost, and structural customizability^36^, aligning well with the design requirements of our catheter system. This approach further supports the development of a low-stiffness catheter architecture, which is advantageous for endoluminal navigation and integrated sensing applications.

### Signal acquisition module for real-time multi-channel readout

**Fig. 3:**
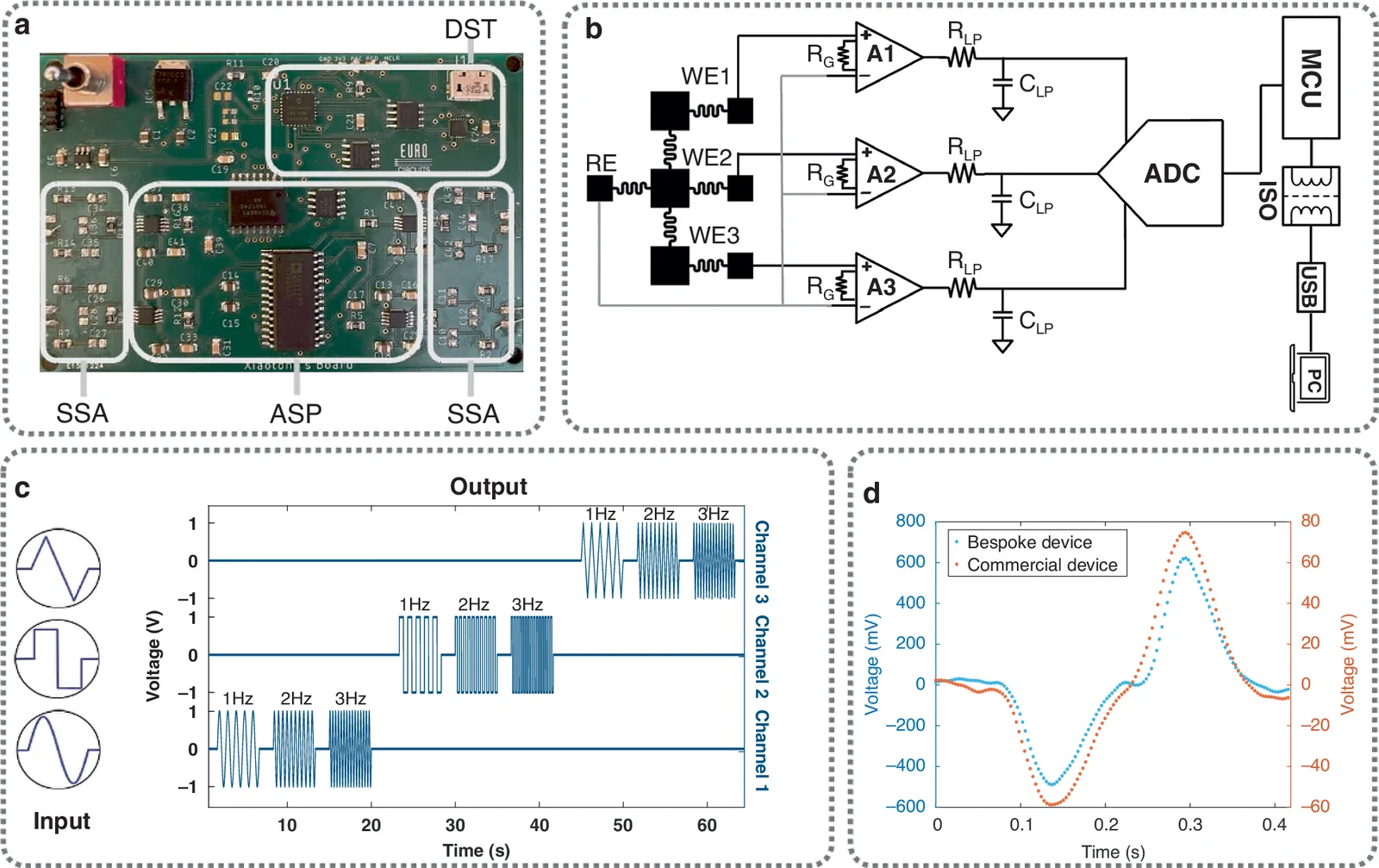
**Customized signal acquisition module for real-time multi-channel readout**. **a** Image of the signal acquisition system board. **b** Circuit design: RE = reference electrode, WE = working electrode, R_G_ = gain resistor of the instrumentation amplifier, R_LP_ = low-pass filter resistor, C_LP_ = low-pass filter capacitor, MCU = micro controller unit, PC = personal computer; and a graph showcases the processed digital output from the circuit. **c** Signal readout characterization from three channels under different waveforms (ramp, square, and sinusoid) with different frequencies applied. **d** Consistency evaluation between the customized module and a commercial recording instrument when measuring the same piezoelectric sensor output

The customized signal acquisition module was developed to support real-time multi-channel readout of the catheter-integrated sensor array during catheter advancing with withdrawal. The impedance of piezoelectric-based sensor is significantly high with over 1 MΩ in low frequency^3^. The module provides high-input-impedance signal conditioning, analogue signal processing, analogue-to-digital conversion, digital signal transmission, and on-screen visualization of individual sensing units. This design matches the electrical characteristics of the P(VDF-TrFE)-based sensors, which exhibit high impedance at low frequencies^3^, and enables multi-channel monitoring during catheter interventions. Together, these functions allow the sensor array, catheter body, and display interface to operate as an integrated pressure feedback system. As shown in Fig. 3a, the system consists of four parts, sensor signal acquisition (SSA), analog signal processing (ASP), digital signal transmission (DST) and real-time display (RTD). And the signal acquisition circuit used in this work is a custom-designed architecture developed for multi-channel piezoelectric sensing, and the detailed circuit schematic is shown in Fig. 3b. A flowchart showcasing the detailed structure of the system is shown in Fig. S14.

To test the signal acquisition system, different waveforms of variable frequencies generated from a function generator are fed into three input channels (C1, C2, and C3) of the circuit board in sequence, and each channel signal is plotted out over time correspondingly, as shown in Fig. 3c. In detail, channel one first receives 1 Hz, 5 Hz and 10 Hz sinusoidal signals in sequence while the other two channels remain near-zero baseline. Then square and ramp waveforms are received from channel two and three independently in turn. These results illustrate the circuit’s ability to independently acquire signals from multiple channels without interference and visualize the results in a real-time display. Furthermore, the performance of our acquisition system exhibits close concordance with that of a commercial signal recording instrument (MSO-X 3054 A) when measurements are conducted directly on a single sensing element of the piezoelectric sensor (Fig. 3d).

### Sensor characterization

**Fig. 4:**
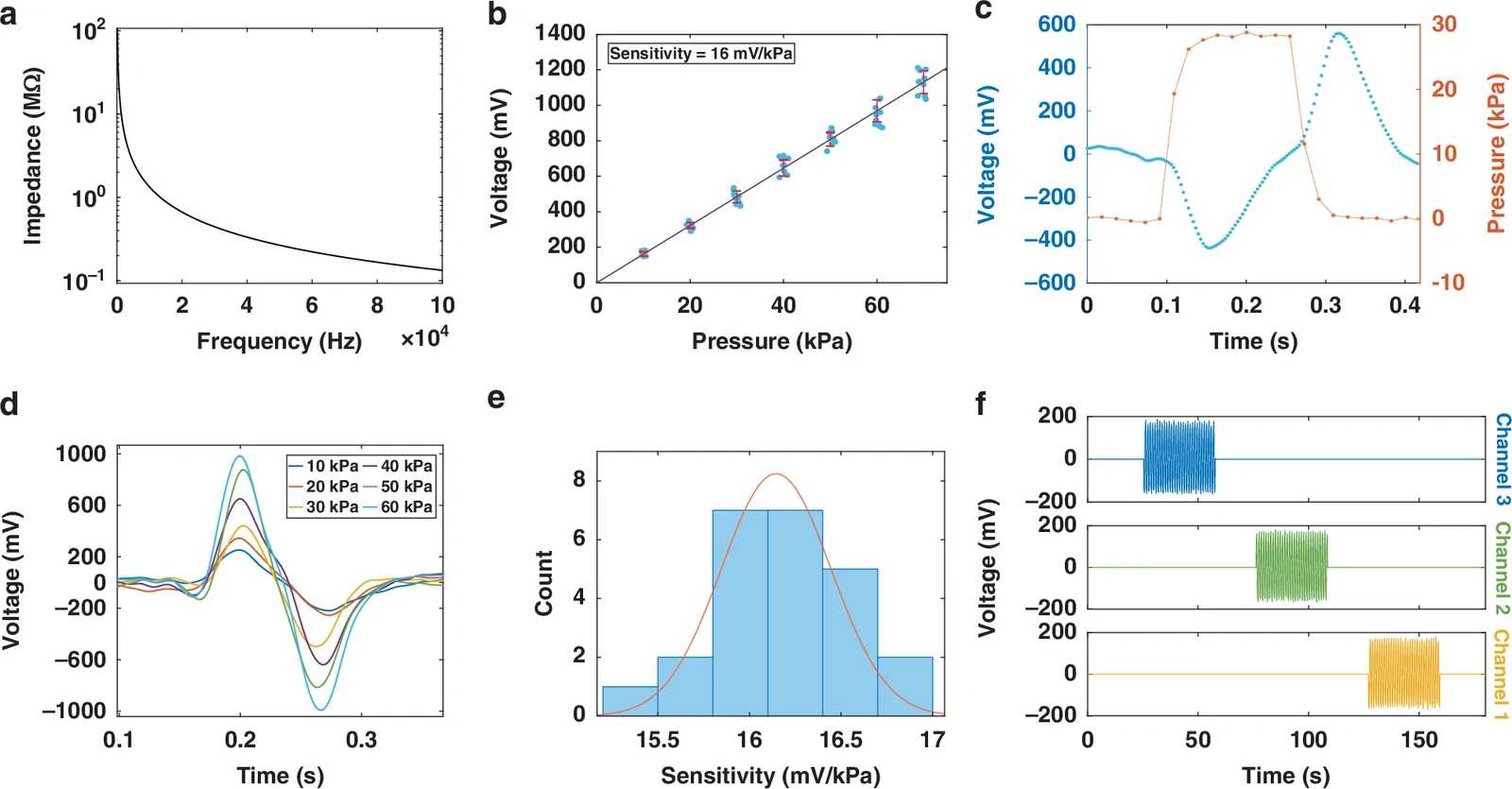
**Characterization of the sensor array**. **a** Semi-logarithmic plot demonstrating impedance variance of the sensor across different frequencies measured with commercial impedance analyzer. **b** Output peak voltage corresponding to various ranges of pressure. **c** Voltage response to a single cycle of force loading and unloading. **d** Voltage response corresponding to various ranges of pressure. **e** Histogram and approximated Gaussian line of the sensitivity measurements across multiple sensing units. **f** Force application with a repeated loading and unloading cycle on the sensor array

The impedance, sensitivity, voltage response, and multiplexing capability of this sensing system were characterized. The impedance of a single sensing unit was measured using an impedance analyzer (model E4990A, Keysight Technologies, CA) across a frequency range from DC (direct current, 0 Hz) to 100 kHz, as illustrated in Fig. 4a. The sensing unit exhibited an impedance exceeding 10 MΩ at low frequencies, which is characteristic of a typical flat piezoelectric transducer and highlights its capacitive nature.

The sensing units and the multiplexed sensor array were characterized with our bespoke recording system. An experimental setup using a bespoke force application stage is demonstrated in Fig. S9. As shown in Fig. 4b, the sensor demonstrates a high linearity in voltage output across a typical range of intrabody pressures (0–80 kPa), with 10 repeated cycles conducted at each pressure level (*n* = 10). This aligns with many catheter-based surgical procedures, for instance, catheter ablation, pressure from 0 to 26 kPa for a hemispherical catheter tip^40^. Its sensitivity (*S*) is determined by the equation *S* = *δV/δP*, where *V* is the measured voltage at pressure *P*. Although the nature of high impedance, the sensor supported by our bespoke data acquisition system exhibits a 25 times higher sensitivity of 16 mV/kPa, compared with the state-of-the-art catheter integrated pressure sensors, as shown in Table S1. The voltage response is sensitive to the dynamic change of pressure (force loading and unloading) and demonstrates a negligible time delay under a loading rate of 0.5 kPa/ms, as shown in Fig. 4c. The observed voltage response further demonstrates consistent and reproducible signal morphology across successive force-loading cycles under varying pressure conditions (Fig. 4d), characterized by a distinct positive peak during loading and a corresponding negative peak during unloading. This behavior aligns with the principles of piezoelectric transducers subjected to dynamic mechanical stimuli, wherein the increase of pressure induces charge displacement toward the sensor’s cathode, while the decrease of pressure generates a symmetric charge displacement in the opposite direction.

To evaluate the reproducibility in sensitivity, eight sensing arrays with a total of 24 sensing units were fabricated and individually characterized (*n* = 24). The result presents an average of 16.1 mV/kPa and standard deviation of 0.7 mV/kPa under Gaussian approximation (Fig. 4e). Uniformity of output signals in multiplexing was tested by sequential force application at a pressure of 10 kPa and loading cycle frequencies of 1 Hz on different sensing units, as shown in Fig. 4f. Signals recorded in each 30 s long loading cycle reveal minor variance in morphology and amplitude. Negligible crosstalk was observed on the uncontacted channels. Noise was filtered with the built-in analogue and digital filters in our recording system.

### Catheter multi-directional sensing study

The in-vitro validation was designed to progress from controlled quantitative benchmarking to anatomically inspired scenario testing. A straight 3D-printed conduit with periodically arranged protrusions was first used to evaluate the directional sensing and spatial localization capability of the multiplexed sensor array under well-defined geometric constraints. Subsequently, a soft aortic arch model and a branched bronchial model were employed to assess the device response in curved, confined, and branched endoluminal geometries that more closely approximate representative intervention pathways.

**Fig. 5:**
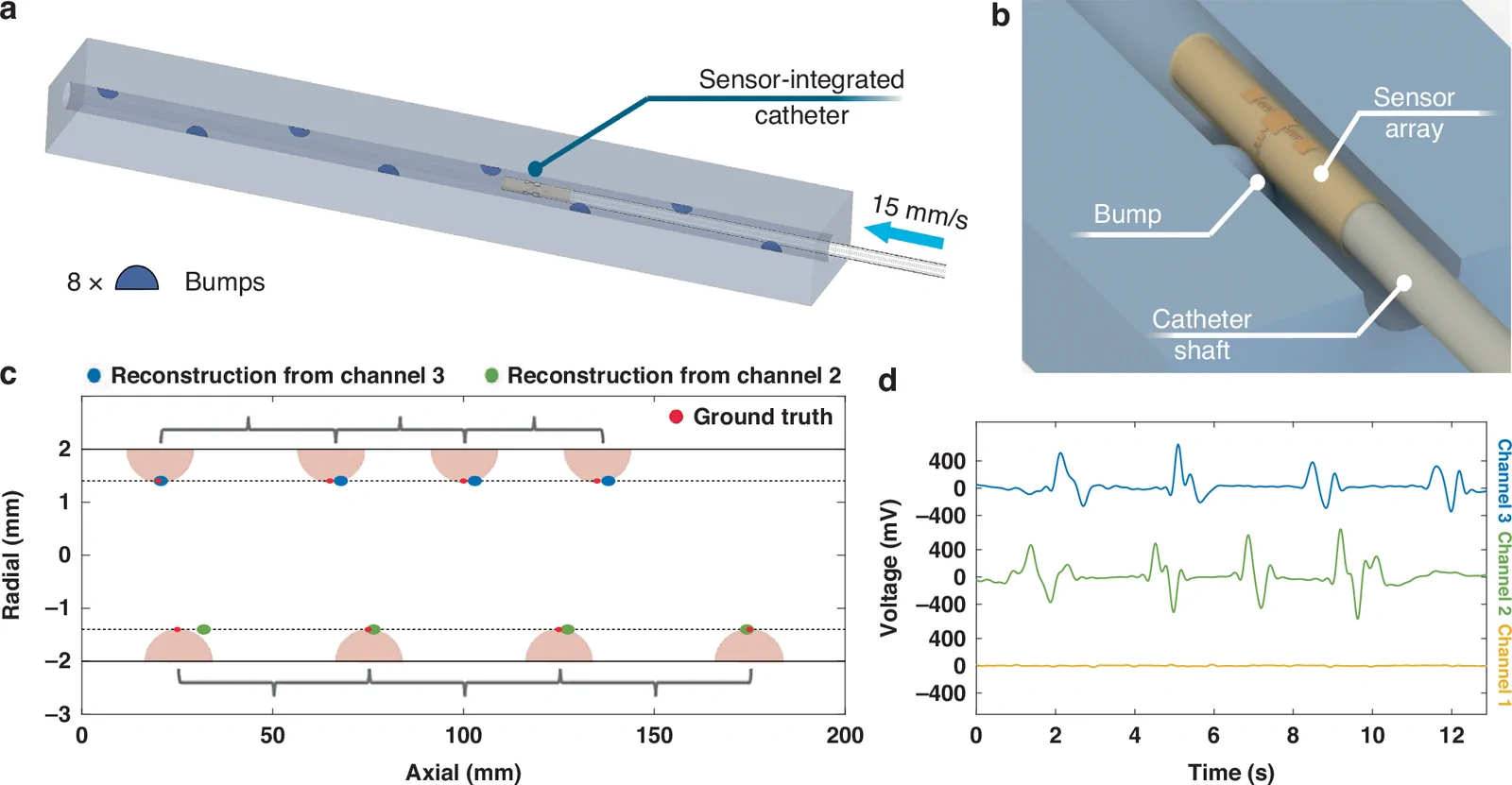
**Controlled benchmark study of catheter multi-directional sensing and spatial localization**. **a**, **b** Schematics of the 3D-printed conduit model with periodically arranged protruding bumps used for quantitative evaluation under well-defined geometric constraints. **c** Reconstruction of in-contact locations of individual bumps from the recorded pressure peaks and comparison with ground-truth bump positions. **d** Multi-channel sensor output signals recorded during catheter insertion

The multiplexing performance of the sensor array upon assembling with the catheter was conducted on a 3D-printed model of a general biological conduit. To test the directional sensing of the device, the catheter was inserted into this conduit model where four pairs of protruded bumps were distributed with designated distances on the inner wall (Fig. 5a). As the catheter navigated through the conduit, the sensor array measured pressure signals from two directions where the catheter body was in physical contact with the protruding bumps (Fig. 5b). During the experiment, the delivery speed in catheter insertion at the proximal end remained constant at 15 mm/s with a motorized linear stage. Given the multi-directional pressure monitoring enabled by the sensor array at the catheter’s distal end, contact reconstruction, defined here as the estimation of catheter–tissue contact locations based on the pressure signals recorded by multiple sensing units, can be used to infer the spatial locations of tissue contact. Figure 5c demonstrates the reconstruction of contact locations according to the recorded pressure peaks when the catheter passed each bump sequentially (Fig. 5d), showcasing an accurate estimation with an average spatial uncertainty of 2.2 mm. No crosstalk disturbance was observed at the uncontacted sensing unit. Additionally, we conducted a finite element analysis (FEA), confirming that the pressure on the sensing area increased proportionally with compression displacement during the bump contact (Fig. S15).

### Pre-clinical studies

**Fig. 6:**
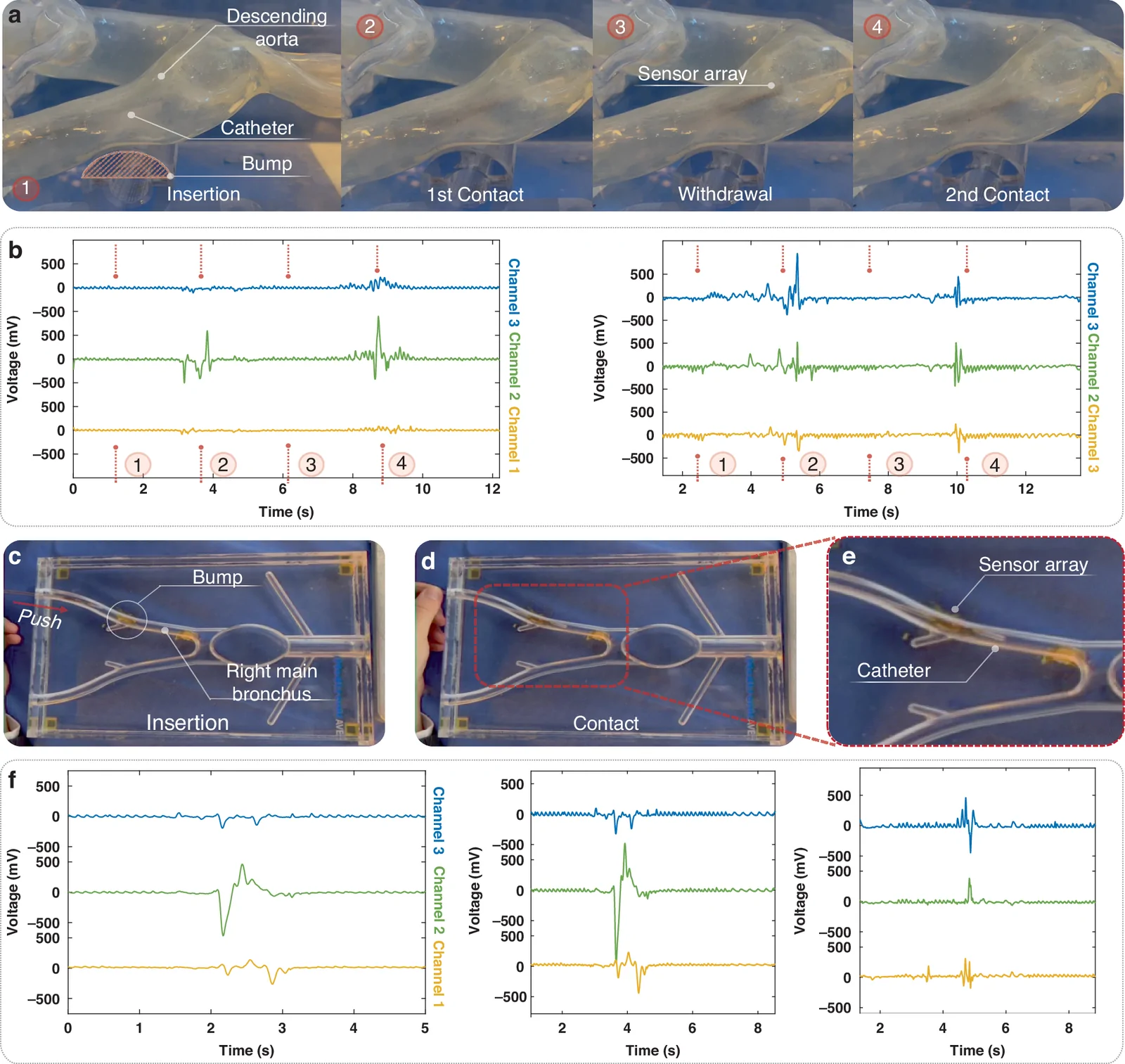
**Results of in-vitro phantom studies**. **a** Sequential images showing catheter insertion and withdrawal within an aortic arch phantom containing an artificial bump. The labels in red balloons indicate the temporal sequence of the images. **b** Two representative examples of sensor output signals recorded during the aortic arch phantom study. The timestamps corresponding to the images are labeled in red. **c**, **d** Illustrations of catheter manipulation during navigation through a simplified bronchial phantom with a small protruding bump located at the midpoint. **e** Magnified image showing the catheter in contact with the bump. **f** Representative sensor array readouts

To complement the controlled straight-conduit benchmark, the integrated device was further evaluated in anatomically inspired in-vitro phantoms with curved and branched geometries. Specifically, a soft aortic arch phantom was used to represent a curved vascular pathway, while a branched bronchial phantom with a localized narrowing was used to represent confined airway navigation. The selection of the phantoms was based on the typical catheter-based surgeries, i.e., endovascular and bronchial interventions. The in-vitro experiment on endovascular interventions started by navigating the catheter through the arch of aorta, with access via the descending aorta through an introducer. The catheter was then gently moved back and forth through the vessel multiple times Fig. 6a. A distinct signal peak was observed at sensor 2, positioned on the catheter at the site of physical contact with the synthetic protrusion (Fig. 6b, left). Smaller signal peaks were also recorded in the remaining sensors, likely attributable to contact between the catheter and the narrow vascular passage. This procedure and the sensor measurement were simultaneously recorded as shown in Supplementary Video S1. The right plot in Fig. 6b shows another set of measurements in which sensor 2 and sensor 3 were in contact with the protrusion. The complex shape of signals reveals the complicated and dynamic interactions between catheter and tissues.

An in-vitro study on a simplified bronchial phantom was conducted to illustrate the feasibility of the device in the clinical scenario of bronchial and lung interventions. Figure 6c–e showcases the catheter navigated through the right bronchi and passed a narrowed down air passage, representing bronchial inflammation or tumor. The sensor array recorded a significant negative spike of −0.6 V on the sensor in direct contact with the bump, representing an instant increase of pressure to around 40 kPa (Fig. 6f). While the other sensing units in contact with the wall of bronchi were observed minor and discontinuous amplitude shifts. This showcases the sensor array can detect the direction of major dynamic physical contact while retaining the information of minor physical contact in other directions. The procedure and corresponding sensor measurements are shown in Supplementary Video S2.

To analyze the clinical interpretation of the in vitro measurements, we reviewed the reported contact pressure and interference from physiological motions in endoluminal interventions (Table S3). In endovascular environments, the main dynamic interference arises from vascular pulsation, which produces pressure variations of ~5.3 kPa at 60–120 cycles min⁻¹, whereas diastolic blood pressure is less relevant to the present piezoelectric sensing mechanism because the sensor primarily responds to dynamic force. In bronchial environments, respiratory motion typically generates airway pressures of 1.5–3.9 kPa at 12–20 cycles min⁻¹. Based on these values, we defined practical detection thresholds of 6 kPa for endovascular interventions and 4 kPa for bronchial interventions to distinguish tissue–catheter contact from physiological background motion. In the pre-clinical experiments, the sensor array recorded peak voltage responses of approximately 0.3–0.6 V, corresponding to contact pressures of approximately 20–40 kPa, which are higher in folds than the detection thresholds. In addition, the FEA simulation showed that even a minimal contact displacement of 0.5 mm generated a pressure exceeding these thresholds (Fig. S15). These results indicate that the sensing system can detect localized tissue–catheter interactions, such as contact with protrusions or other anatomical features, with sufficient contrast against representative physiological motion-induced interference in both endovascular and bronchial scenarios.

## Discussion

The multiplexed catheter-integrated pressure sensing system developed in this work demonstrates how soft piezoelectric materials, scalable fiber drawing, and miniaturized electrode design can be combined to create a clinically meaningful platform for intraluminal pressure monitoring. A central contribution of this study is the coordinated optimization of the sensing material, catheter structure, and signal acquisition circuitry. This co-design approach enables multiaxial pressure feedback without sacrificing device flexibility, catheter profile, or compatibility with standard interventional instruments. The sensor achieved sensitivity of approximately 16 mV per kPa, with a reproducibility characterized by a standard deviation of only 0.7 mV per kPa, surpasses existing catheter-integrated pressure sensors by a factor greater than ten. This enhanced performance results from both the intrinsic piezoelectric characteristics of P(VDF-TrFE) and the optimized thin-film deposition, compliant electrode pattern, and high-impedance acquisition architecture introduced in this study. The piezoelectric sensing mechanism enables self-powered dynamic force detection, while the sandwich electrode structure with radially symmetric multiplexing allows omnidirectional sensing on the catheter shaft. In addition, the integration of the sensor array with a catheter shaft enables circumferential pressure detection for catheter-based interventions.

The thermal drawing method used to fabricate the catheter is another significant advantage. Unlike extrusion or traditional assembly methods, thermal drawing preserves micro-scale lumen geometry along fibers that can reach several meters in length. This uniformity supports organized wire routing and reduces strain gradients in the distal sensing region. The scalability of this fabrication technique is beneficial for clinical translation, since it allows rapid prototyping of customized catheters tailored to a variety of anatomical locations or multifunctional surgical tasks. In addition, the multi-lumen configuration provides design flexibility. Extra lumens may accommodate optical fibers, microfluidic channels, steerable components, or therapeutic tools, which opens the possibility of fully integrated interventional systems based on a single catheter platform.

The in vitro experiments further highlight the potential clinical relevance of the system. In the biological conduit model, the pressure signals recorded from the multiplexed sensor array enabled reconstruction of contact locations with a spatial uncertainty of 2.2 mm. This demonstrates that dynamic pressure feedback can reveal structural features along a lumen, even when imaging support is limited. In the aortic arch and bronchial phantoms, the system distinguished major protrusions from background wall interactions, reflecting realistic scenarios such as aneurysmal bulges, airway stenosis, or localized tissue thickening. The ability to identify such contacts may assist clinicians during navigation, help prevent excessive force on vulnerable tissues, and potentially support robotic or semi-autonomous navigation strategies that rely on real-time pressure data. While the present in-vitro studies demonstrate directional sensing in controlled and anatomically inspired phantoms, future studies will incorporate dynamic physiological motion and in vivo evaluation to further assess performance under more complex biological conditions. In particular, in vivo studies will be essential to validate the device’s functionality in realistic tissue environments, where factors such as tissue heterogeneity, biological fluid interactions, and organ motion may influence sensing accuracy and stability. Feedback from these in vivo evaluations will further inform iterative optimisation of the catheter design for future clinical translation.

Several minor limitations should be acknowledged, although none of them affect the central conclusions or diminish the advantages demonstrated. The current prototype uses a three-channel sensing array, which is sufficient to validate multi-directional detection. Higher-density arrays can be implemented easily due to the modular electrode design and the multi-lumen catheter structure. The signal acquisition system presently operates as an external module, which is typical for early-stage systems. Future versions can be miniaturized without altering the sensing principle. Testing was performed in controlled phantom environments, and although biological tissues show more variation, the compliant design of the sensor is expected to adapt well to clinical conditions. Catheter bending may introduce small baseline variations, but these remain minor due to the serpentine electrode layout and PTFE encapsulation. Significant slow-loading cycles can produce slight hysteresis due to the dynamic nature of piezoelectric materials. However, endoluminal interactions are typically transient, so this behavior has negligible impact on practical use. Practical deployment may also require attention to sterilization and repeated cleaning, as thermal, chemical, or mechanical exposure could gradually affect encapsulation integrity and long-term wiring reliability. Although the current materials and packaging approach are intended to be robust, additional validation under clinically relevant sterilization and reprocessing conditions will be important to confirm durability over repeated use.

In summary, a multiplexed catheter-integrated pressure sensing system has been developed for digitalizing tissue interaction during endoluminal interventions to minimize tissue damage and enhance surgical outcomes. The pressure sensing system consists of a miniaturized and flexible piezoelectric sensor array, a bespoke medical catheter hosting signal transmission and instrument deployment, and a signal acquisition module optimized for piezoelectric-based sensing materials. The sensor array is fabricated from a thin P(VDF-TrFE) film with patterned electrode layers, forming a compact and configurable layout that supports conformal attachment to the curved catheter surface. To accommodate the electrical characteristics of the P(VDF-TrFE)-based sensor, the signal acquisition module is designed and optimized, achieving real-time signal readout and visualization. On top of it, the sensor demonstrates a higher sensitivity and a comparable sensing range, compared to the state-of-the-art catheter-integrated pressure sensors. The medical catheter platform is built to equip with multiple functional lumens and demonstrates seamless sensor integration and instrument deployment therein, facilitated by the highly scalable and low-cost thermal drawing technique. The integrated system exhibits high sensitivity in detecting pressure variations originating from multiple directions across the catheter surface. The in-vitro phantom studies further highlight the multi-directional sensing within confined anatomy. These capabilities can provide clinicians with real-time feedback on catheter–tissue interactions, potentially improving navigation safety and reducing the risk of unintended tissue damage during minimally invasive procedures. For device engineers, the modular fabrication strategy and scalable catheter platform offer a flexible approach for integrating sensing functions into next-generation endoluminal medical devices. In general, the proposed multiplexed pressure sensing system enables a circumferential and rapidly prototyped catheter-based solution for pressure feedback in endoluminal interventions.

## Methods

### Fabrication and integration workflow

The overall fabrication and integration process of the catheter-integrated sensing system is illustrated in Fig. S1. The workflow follows a modular approach that includes sensor fabrication, multi-lumen catheter thermal drawing from a 3D-printed preform, sensor–catheter integration, and final system assembly with the signal acquisition module.

### Sensor fabrication

The pressure sensor was constructed by encapsulating a P(VDF-TrFE) thin film between two metallic electrode layers composed of Au/Cr. The polymer layer was prepared via spin-coating. Specifically, P(VDF-TrFE) was dissolved in dimethylformamide at a concentration of 1%, and the resulting solution was spin-coated onto an atomically flat, highly conductive Si substrate at 2000 rpm for 30 s. Following ambient drying, the film was annealed at 140 °C for 10 min to promote crystallization. The annealed film was subsequently electrically poled at 23 °C under an incrementally increasing alternating-current voltage. Electrode patterning was achieved using stainless-steel masks (100 µm thickness) fabricated via laser cutting, as shown in Fig. S2. A 2–3 nm Cr adhesion layer was first deposited by thermal evaporation, followed by thermal evaporation of a 100 nm Au layer at a rate of 2 Å/s, yielding a smooth and well-defined electrode surface.

### Custom signal acquisition circuit configuration

Within the ASP module, the weak signals generated by the piezoelectric sensor are conditioned using high-input-impedance instrumentation amplifiers (INA116), followed by low-pass filtering (LPF) and analog-to-digital conversion (ADC). The digitized signals are transmitted through the SPI interface to the MCU in the DST module for data encoding and communication, and subsequently sent via UART to the RTD module, where an isolator and USB interface enable safe data transfer to a computer for visualization and recording.

### Sensor-catheter assembly

The assembly began with cutting the sides of the fiber to expose the side lumens, gaining access for the conductive wires. The electrode pads for anodes and ground of the sensing elements were then connected with the pull-our wires via conductive Cu pads (Fig. S5a). Subsequently, the wires were fed into their corresponding lumens until reaching the preassigned position (Fig. S5b). After that, the sensor was gently bent and wrapped over the distal end of the fiber. The process was finalized by wrapping a layer of PTFE tape (100 μm thickness) over the entire sensor and corresponding wires, as a protective layer preventing sensors from scratch damage and short circuit caused by body fluids.

### Fiber drawing

The fiber preform was first conceptualized using 3D computer-aided design software (SolidWorks, Dassault Systèmes, France) and fabricated using a fused-filament fabrication 3D printer (Ultimaker, PC Transparent, 2.85 mm; Netherlands). The preform allows for consistent reproduction of fine structural features required for subsequent thermal drawing. This fiber preform was then placed in a vacuum oven maintained at 70 °C for 48 h to effectively dehydrate and eliminate residual moisture content that could compromise thermal stability, induce bubble formation, or introduce defects during consolidation.

The fiber drawing process was initiated by securing the prepared preform onto a vertically aligned linear motorized stage positioned above a three-zone cylindrical heating furnace. Once the temperature at each zone of the furnace reaching 150 °C, 230 °C, and 85 °C (from top to bottom), the linear stage continuously fed the fiber preform into the furnace at a constant speed of 1 mm/min (*V*_*feed*_). After the polymer preform began to neck down, a downward pulling force was applied from the bottom side using a speed-controllable capstan (*V*_*draw*_). The resulting diameter of the fiber (*D*_*fiber*_) can be approximated using the following equation:

1 $$\frac{{{D}_{{fibre}}}^{2}}{{V}_{{draw}}}=\,\frac{{{D}_{{preform}}}^{2}}{{V}_{{feed}}}$$

Meanwhile, the diameter of the fiber was continuously monitored using a laser measurement device, which provides real-time feedback for the operator to adjust the capstan speed, thereby controlling the fiber diameter. Finally, fibers with desired diameter were cut into sections with the targeted length for catheter preparation.

### Catheter stiffness characterization

The stiffness of the catheter was evaluated by conducting a flexural rigidity test. The distal end section, where the sensor mounted, was tested with wires embedded. The experiment involved vertically displacing the catheter tip (50 mm) by 10 mm deflection, repeated over three trials. A commercial force sensor was used to measure the overall force applied (Nano43, ATI Industrial Automation, USA). The stiffness is calculated with the total force required for different amplitudes of tip deflection and expressed in N/mm.

### Sensor array characterization

The characterization of sensor array was performed on a bespoke platform for force loading and unloading with a controllable cycle frequency, as shown in Fig. S9. The contact area was determined via a customized spring probe mechanism (Fig. S10).

The sensitivity of individual sensors was tested by repeatedly applying cycles of various pressures. The effective pressure in each cycle was derived from equation *P* = *F/A*, where *F* is the peak force from the scale and *A* is the surface area of the probe tip. Pressures ranging from 10 kPa to 70 kPa with a step of 10 kPa were experimented. 10 repeated cycles were conducted for each pressure. The voltage output from the sensor was determined by the positive peak in each loading cycle. Due to the high linearity, a first-order polynomial fitting was used for sensitivity calculation.

### Biological conduit test

The mechanical performance of the catheter-mounted sensor array under multidirectional loading was systematically evaluated using a custom-designed experimental platform incorporating a 3D-printed biological conduit (Fig. S11). This conduit model was constructed to replicate the geometric complexity and luminal irregularities typically encountered in physiological environments, enabling controlled and reproducible assessment of sensor response. During testing, the catheter was advanced exclusively with an axial insertion force, ensuring that the contact force exerted on the sensor array remained consistent as the device traversed each predefined bump along the conduit wall. To maintain a constant and repeatable insertion velocity, the catheter was secured onto a precision linear translation stage driven by a programmable motor. The spatial arrangement of the raised features within the conduit ensured that only two lateral regions of the sensor array were engaged in direct mechanical contact, while the remaining sensing elements experienced minimal or no applied pressure. This controlled asymmetry in loading conditions allowed for isolated characterization of directional sensitivity and crosstalk within the array. Overall, this experimental setup provided an accurate and reproducible means of quantifying the ability of the sensor to detect localized mechanical events during catheter navigation.

### In-vitro phantom study

#### Endovascular interventions

A 1:1 life-sized soft silicone anatomical model of the aortic arch featuring a physiologically accurate three-cusp inflow region (T-S-N-002-v2, Elastrat, Switzerland) was employed to assess the sensorized catheter’s behavior under conditions representative of clinical endovascular procedures (Fig. S12). The experimental protocol began with the insertion of the catheter into the phantom through a unidirectional valve, which served to replicate the functional characteristics of a standard catheter introducer used in vascular access. Once positioned within the phantom, the catheter was advanced and retracted along the curvature of the vascular wall to evaluate the fidelity of sensor–wall interactions, with particular emphasis on detecting contact events with both the endovascular surface and the embedded artificial protrusion designed to simulate pathological irregularities.

#### Bronchial and lung interventions

For respiratory system simulations, a simplified yet anatomically relevant bronchial passage model (Medtronic, US) was utilized (Fig. S13). The catheter was initially introduced parallel to the primary bronchial conduit to mimic navigation through branching airways as encountered during bronchoscopy-guided interventions. Subsequent controlled movement of the catheter along the lumen enabled systematic evaluation of sensor response upon encountering an artificial protruded bump integrated into the model.

## Supplementary information

Revised Supplementary Information

VideoS1

VideoS2
