# Supplementary material for: Multiplexed catheter-integrated pressure sensing system for endoluminal interventions
Source: Microsyst Nanoeng. 2026 Sep 15;12:319. doi: 10.1038/s41378-026-01411-0 (PMC13578360; doi:10.1038/s41378-026-01411-0)
Supplement: Supplementary file 1 [file 41378_2026_1411_MOESM1_ESM.docx]

**Supplementary Information**

**Multiplexed Catheter-Integrated Pressure Sensing System for Endoluminal Interventions**

Xiaotong Guo^1^, Qindong Zheng^2^, Jinshi Zhao^3^, Bing Li^4^* and Eric M. Yeatman ^1, 5^*

^1^Department of Electrical and Electronic Engineering, Imperial College London, London, UK.

^2^Department of Bioengineering, Imperial College London, London, UK.

^3^Department of Metabolism, Digestion, and Reproduction, Imperial College London, London, UK.

^4^Institute for Materials Discovery, University College London, London, UK.

^5^College of Science and Engineering, University of Glasgow, Glasgow, UK.

*Email: e.yeatman@imperial.ac.uk.

**List of Figures**

**Fig. S1**  Fabrication and integration workflow of the catheter-integrated piezoelectric sensing system

**Fig. S2**  Schematic illustration of the multilayer catheter-integrated sensor array

**Fig. S3**  Electrode layout and geometric dimensions of the flexible sensor array

**Fig. S4**  Comparison of sensor voltage with the negative pressure derivative

**Fig. S5**  Photographs of catheter–sensor integration and wiring

**Fig. S6**  Flowchart of the signal acquisition board process

**Fig. S7**  Rising and falling edge transient responses of the filtered signal

**Fig. S8**  Filtered square-wave output demonstrating signal integrity

**Fig. S9**  Characterization setup schematic for force-loading validation

**Fig. S10**  Spring probe design and tip configurations for force application

**Fig. S11**  Design of the biological conduit experimental setup

**Fig. S12**  Vasculature phantom used in in-vitro evaluation

**Fig. S13**  Bronchial air passage phantom with artificial bumps

**List of Tables**

**Table S1** Comparison of catheter-integrated pressure sensors with previous work

**Table S2** Typical sizes of human lumens in different endoluminal interventions

**System Fabrication Flowchart**

**Fig. S1** Fabrication and integration workflow of the catheter-integrated piezoelectric sensing system, including sensor fabrication, multi-lumen catheter thermal drawing, sensor–catheter integration, and final system assembly.

The fabrication workflow adopts a modular, layer-based strategy that separates sensor fabrication, catheter fabrication, and system integration into independent steps. This modular approach enables rapid prototyping because each component can be fabricated, tested, and modified individually without redesigning the entire system. For example, the electrode layout and sensing geometry can be easily adjusted through shadow mask patterning, while the catheter structure can be customized by modifying the 3D-printed preform used for thermal drawing. As a result, microstructures such as electrode arrays, serpentine interconnects, and lumen geometries can be tailored to accommodate different sensing configurations and device requirements. This flexibility facilitates efficient integration of sensing elements into catheter-scale devices and allows the platform to be adapted for various endoluminal applications, supporting both sensor integration and the deployment of medical instruments within the catheter architecture.

**Sensor Fabrication**

P(VDF-TrFE) with a 70:30 molar ratio was purchased from Piezotech (France) and all other chemicals in this study were purchased from Sigma-Aldrich (U.K.). Printed circuit board (PCB) was fabricated and purchased from Eurocircuits (Belgium).

**Fig. S2** Schematic illustration of the catheter-integrated sensor array, including (a) the top-layer serpentine interconnect and sensing elements, (b) the bottom-layer interconnect arrangement, and (c) the combined multilayer design showing the complete sensor structure.

The pressure sensor was fabricated by sandwiching a thin film of P(VDF-TrFE) between two deposited layers of Au/Cr composition. This fabrication process was achieved by a spin-coating technique. Dimethylformamide (DMF) with 1% concentration rate was initially used to dissolve P(VDF-TrFE). The resultant solution was spin-coated, in 2000 rpm for 30 s, onto a highly conductive and atomically flat Si substrate to form a P(VDF-TrFE) thin film. After being dried at room temperature, the film was then annealed at for 10 min at 140 ℃ to enhance crystallinity. Eventually, as described in previous report ^1^, the film was poled electrically under increasing Alternating current (AC) voltage at 23 ℃. Patterned masks were shaped by a high precision 4-axis laser cutting system (Rofin, Germany) from stainless-steel foils with 100 μm thickness. The adhesion was a 2-3 nm thick thermal evaporated chromium (Cr) layer. A 100 nm Au film was evaporated thermally with a deposition rate of 2 A/s onto the adhesion layer, resulting in a flat electrode surface.

**Fig. S3** Electrode layout and geometric dimensions of the flexible sensor array.

The electrode layout is designed to support high-density integration while maintaining mechanical compliance for catheter deployment in curved luminal environments. The sensing electrodes are arranged in a compact array with short interconnection distances, allowing multiple sensing units to be integrated within a limited catheter footprint. Serpentine interconnect traces are introduced between the electrode pads to provide mechanical stretchability and strain relief. This geometry effectively distributes mechanical stress during bending, twisting, or catheter manipulation, preventing stress concentration at the electrode junctions. As a result, the sensing elements can conform to curved lumen surfaces while maintaining stable electrical connections. In addition, the widened electrode pads improve contact reliability and reduce electrical resistance, which helps preserve signal integrity during deformation and repeated contact events. Together, this electrode configuration enables dense sensor integration, mechanical adaptability to complex anatomical geometries, and reliable signal acquisition under dynamic loading conditions.

**Sensor Dynamic Response Verification**

To verify the sensing mechanism, the measured voltage signal was compared with the negative time derivative of the applied pressure. The PVDF-TrFE sensor output exhibits a strong correspondence with the temporal profile of the pressure’s rate of change rather than with its absolute amplitude. This behavior aligns with the intrinsic characteristics of piezoelectric polymers, which generate electrical signals predominantly in response to dynamic mechanical stimuli, such as variations in stress or strain. Rapid changes in applied force produce transient voltages proportional to the force derivative, whereas sustained loads result in rapid signal decay due to charge leakage and internal relaxation processes. The close agreement between the sensor voltage and the pressure derivative therefore confirms that the sensing unit is highly responsive to transient mechanical events and exhibits limited sensitivity to static or slowly varying forces.

**Fig. S4** Comparison of the PVDF-TrFE sensor voltage output with the negative time derivative of the applied pressure.

**Sensor on Catheter Integration**

**Fig. S5** Integration of sensor on a bespoke catheter. (a) Image of the sensor-catheter integration. (b) Image demonstration of the wire connections linking the catheter channels to the sensor.

Integration of sensor on the thermally drawn fiber requires a tight sensor attachment onto the fiber’s outer surface while maintaining a stable wire connection. Therefore, the fiber is designed and fabricated with multiple channels for accommodating wires. The wires are then post-fed into the channels individually and exposed via small window openings on the outside of the fiber. These window openings are cut by micro-blade. Each sensor electrode is soldered with individual wire to provide a secure electrical connection. This internal wiring within the catheter prevents interference between different sensing units and ensures a reliable connection between the sensor and the test circuit board at another end of the catheter. Also, internal wiring avoids noise interference due to wire exposure and contact with tissues.

**Signal Acquisition System**

The system comprises four main functional modules: Sensor Signal Acquisition (SSA), Analog Signal Processing (ASP), Digital Signal Processing (DSP), and Real-Time Display (RTD). The SSA block captures raw analog signals from individual sensors. These signals are conditioned in the ASP stage, which includes an instrumentation amplifier (IN-AMP), a low-pass filter (LPF), and an analog-to-digital converter (ADC) to enhance signal fidelity and suppress noise. The digitized signals are then transmitted to the DSP module, where a microcontroller (MCU) performs data packaging and manages serial communication protocols. The processed data are transmitted to a host computer via a USB interface for real-time display, storage, and visualization. A dedicated power supply system supports all modules with regulated voltages, ensuring signal integrity and system stability.

The data acquisition circuit presented is tailored designed for our multiplexed force sensor, providing high-precision signal conditioning, filtering, digital conversion and transmission for dynamic force measurements. Each sensing unit output is connected to an instrumentation amplifier (INA116), labelled as A1 to A3, which provides high input impedance to accommodate the high impedance of the sensor and high differential gain to amplify the low-level signals of the sensors. Connecting each working electrode (WE1 to WE3) to a separate amplifier allows independent signal conditioning and minimizes crosstalk between channels, improving measurement accuracy and reliability. After amplification, each channel’s output is connected to a low-pass filter consisting of a resistor (R_LP_) and capacitor (C_LP_). These filters attenuate high-frequency noise and prevent aliasing, preserving signal integrity for accurate force measurement. Following filtering, the conditioned analogue signals are converted to discrete digitized signals with high resolution through an analogue-to-digital converter (ADC, AD974).

A 16-bit microcontroller (PIC24FJ64- GA002) is used to control data acquisition time, process the digitized data and achieve signal transmission via a microchip kit (PICkit 3 programmer) to the USB port of a computer. Both microcontroller and the PICkit 3 programmer can be set and modified by the MPLAB IDE interface (Integrated Development Environment) with C code configuration. To protect the sensitive analogue circuitry from noise and grounding issues, an isolator (ADUM1201) is implemented between the MCU and the USB interface, mitigating potential interference that may arise when connected to a computer. The data can therefore be displayed in real-time and collected for further analysis using MATLAB. A digital low-pass filter with a cut-off frequency at 10 Hz is then applied to the signal.

**Fig. S6** Flowchart of the signal acquisition board process, illustrating serial port configuration, data buffering, bit-by-bit processing, and data storage operations.

**Fig. S7** Transient response of the filtered output signal. (a) Rising-edge waveform, showing a rapid 10–90% transition within less than 30 ms. (b) Falling-edge waveform, likewise exhibiting a fast 10–90% transition time under 30 ms. These results confirm the system’s ability to track abrupt signal changes with minimal delay.

**Fig. S8** Five consecutive cycles of the filtered square-wave output. The waveform maintains stable amplitude and shape with minimal distortion and only ~1% overshoot, indicating preserved signal integrity following filtering.

**Sensor Characterization Setup**

**Fig. S9** Schematics of the characterization setup compromising labels for the linear force application mechanism and the measurement instrument.

A bespoke experimental setup has been developed for sensor characterization and validation (Fig. S7). The setup was designed to characterize the sensor by repeatedly applying a controllable vertical force on the sensor and comparing the characterization results with a commercial high-precision scale. Also, the setup aims to provide characterization of multi-points force sensing. This setup is mainly composed of the following components: magnetic linear motor (Faulhaber LM038004001), 3-D printed spring probe, measurement plate, high-precision scale, x-y linear stage, and mechanical connection components. The vertical force application on the surface of measurement plate is implemented by the 3-D printed spring probe, which transfers a controllable linear motion, delivered by the magnetic linear motor, into forces. The linear motor, which equips with a position sensor (1 µm precision), enables continuous linear translation of its probe with a certain frequency and cycle duration. Force loading on multiple sites is achieved by configurating the tip design of the spring probe. The scale, which mounted beneath the sensor, allows the validation of sensor characterization and acts as a ground truth. A x-y linear stage is integrated into this setup to precisely adjust the position of the sensor, and thus, each sensing element of the sensor can be tested in serial. Additionally, all the mechanical connection components are 3-D printed, i.e., linear motor holder and connector.

Recording from the scale was collected and transmitted to the same interface of sensor acquisition system via a serial port. Given its sampling rate of 5 Hz, the scale recording was then synchronized with the sensor recording in accordance with their time stamp, thereby providing the ground truth for characterizations.

**Spring Probe Design**

**Fig. S10** Spring probe design for force applications. (a) CAD models of various probe tip configurations designed for multi-point force applications. (b) Transparent view of the spring probe design. (c) Image of the 3D printed probe with spring equipped.

A spring probe is developed and 3D printed to enable force application onto the desired location with a specific surface area, thereby controlling the loading pressure (Fig. S8). The spring probe design enhances multi-position detection accuracy and measurement reliability. The spring, with a stiffness of 0.1 N/mm, was selected in order to apply the controllable force onto the sensing area within the target range of intrabody pressure ^2^. The probe is structured to encapsulate a spring, thereby linear translation delivered by the linear motor can be converted into pressure at the distal end of probe. Four different designs of the probe’s tips were proposed in accordance with the number and distribution of sensing units. The designs cover the objectives of sensor characterization, encompassing single, dual, and triple force loading on the sensing units. For the loading on an individual sensing unit, a block with a 1 mm × 1 mm surface area is located at the distal end of the spring probe. For multiple site pressure loading, the blocks are located with spacing equivalent to the sensor design. This design allows simultaneous measurements of multiple sensing units when force applied from the probe. The internal spring mechanism ensures the probe-to-sensor contact maintains consistency during force applied, minimizing mechanical interference between sensing units.

**Biological Conduit Experimental Setup**

A biological conduit phantom has been designed and fabricated using 3D printing techniques to simulate force contact from various angles and spacing, providing a controlled environment for sensor evaluation. The phantom is constructed as a transparent rectangular block, allowing direct visualization of the internal structure and interactions within the channel. The central feature of the phantom is a straight cylindrical channel with a diameter of 6 mm, designed to accommodate the insertion of a catheter while ensuring consistent experimental conditions.

Within the channel, four pairs of semi-spherical bumps, each with a radius of 3 mm, are strategically positioned along the inner wall to create localized contact points. These bumps are arranged in a diagonal pattern, inducing controlled force interactions as the catheter navigates through the channel. On one side of the channel, the bumps are evenly spaced at 50 mm intervals. On the opposite side, the bumps are placed at varying distances of 35 mm, 35 mm, and 45 mm, creating an asymmetrical distribution that introduces diverse interaction dynamics. The precise configuration of these features is illustrated in Fig. S9.

During experimentation, a catheter is inserted into the channel at a constant speed, allowing for the systematic evaluation of the sensor’s spatial and temporal performance. By comparing the recorded sensor data against the ground truth, we aim to assess the accuracy and responsiveness of the sensor system under controlled mechanical interactions.

**Fig. S11** Design of the biological conduit experimental setup. (a) Schematic depiction of the locations and spacing of the bumps, in which the arrow was positioned toward the direction of catheter insertion. (b) Image illustration of the setup. (c) Lateral image of the setup.

**Vasculature Phantom**

(b)

(a)

**Fig. S12** Vasculature 1:1 scale abdominal soft silicon phantom used in the in-vitro phantom study. (a) Labeled illustration of the anatomic structure of the corresponding phantom components. (b) Side view depiction demonstrating the targeted vessel with an artificial bump built by a 3D printed round head holder.

The soft silicone vascular model shown in Fig. S10 indicates the human aortic arch and major branches, including brachiocephalic trunk, left and right subclavian, vertebral and common carotid arteries. Designed with anatomical fidelity, this in-vitro model provides a realistic environment for endovascular procedures. A cylindrical holder was designed and 3D printed to create an artificial bump that protrudes on the pathway of descending aorta, simulating the irregular folds, clog, or tumor inside the blood vessel. A one-way valve is located at the proximal end of descending aorta, which acts as an introducer and allows the introduction of catheters.

In our experiment, the catheter is designed to be inserted via the proximal one-way valve of the descending aorta and then navigated from the open end of the aorta and passed over the bump. When the catheter interacts with the bump or the vessel wall, the sensors mounted on side of catheter are expected to receive signal recordings of the forces.

**Bronchial Air Passage Bifurcations Phantom**

The phantom study for bronchial interventions is conducted on a simplified bronchial air passage bifurcations model. Although the model (Medtronic, US) is originally designed as a simplified vascular flow phantom, the geometric configuration of this model shares key structural characteristics with the human bronchial air passages. Interpretation of its geometric configuration is shown in Fig. S11 The central conduit and its bifurcations resemble the trachea and main bronchi, allowing for its adaptation in the studies of bronchial catheter interventions. While it does not compromise the full anatomical complexity of the bronchial tree, it provides a controlled environment for analyzing tissue interactions during instrument navigations. Additionally, in the model, multiple artificial bumps (3 mm radius, semi-sphere) are configured on the side of the bronchial wall to represent its heterogeneity in the form of folds or irregular protrusions.

**Fig. S13** Simplified trachea and bronchus phantom with two artificial silicon bumps located at the right main bronchus.

**Signal Acquisition System**

**Fig. S14** Schematic diagram of the signal acquisition and processing system for real-time sensor monitoring.

**Finite Element Analysis**

To evaluate the pressure distribution on the catheter during contact with tissue, finite element analysis (FEA) was conducted using ANSYS R18.2 (ANSYS Corp., USA). A three-dimensional model comprising the catheter and a hemispherical bump was constructed in the simulation environment, as shown in Figure S15a. The catheter cross-section geometry was designed to match that of the fabricated device, with a diameter of 2.8 mm and a length of 10 cm, and polycarbonate (Young's modulus: 2.2 GPa) was assigned as the catheter material. The bump was modelled as a hemispherical structure with a radius of 3 mm, and silicone (Young's modulus: 1 MPa) was assigned as its material to simulate soft tissue. The spherical surface of the hemisphere faces the catheter.

To perform the simulation efficiently and accurately, a sweep mesh method was applied along the catheter for mesh generation. A total of 160 sweep divisions were used along the 10 cm catheter length to ensure sufficiently small and uniform elements. A Static Structural model was used to simulate contact between the hemispherical bump and the catheter. To achieve this, one distal cross-sectional surface of the catheter was defined as a fixed support, while the bump was brought into contact with and used to compress the opposite end of the catheter (Fig. S15b).

Fig. S15c illustrates the pressure distribution on the catheter surface at different degrees of compression between the bump and the catheter. Initially, the spherical surface of the hemispherical bump contacted the catheter at a singularity point, and no force was generated between them. As the bump gradually compressed the catheter, the soft bump began to deform and covered part of the catheter’s surface, meanwhile the catheter bends simultaneously (Fig. S15b). The pressure distribution radiated outward from the centre of the contact region. The simulation results show that, as the bump gradually compressed the catheter, the displacement of the bump was proportional to the maximum and average pressure exerted on the catheter (Fig. S15d). Notably, average pressure refers to the mean pressure applied across a single sensing element area (1.5 mm × 1.5 mm).

**Fig. S15** Finite element analysis of a bump compressing the catheter. (a) The geometric design of the catheter and the bump. (b) The compressive force causes deformation of the bump. (c) A series of images showing the pressure distribution on the catheter under different levels of compression. (d) The relationship between relative displacement and the maximum and average pressure in the single force sensing region.

**Comparison of catheter-integrated pressure sensors**

**Table S1.** Comparison of Catheter-integrated Pressure Sensors with Previous Work

| **Existing Technologies** | This work | A^3^ | B^4^ | C^5^ | D^6^ |
| --- | --- | --- | --- | --- | --- |
| **Sensing Materials** | P(VDF-TrFE) | P(VDF-TrFE) | Au and SiO2 | Si NM | Pressure-sensitive rubber |
| **Methods** | Piezoelectric | Piezoelectric | Metal strain gauge | Piezoresistive strain gauge | Resistive |
| **Power Consumption** | Zero | Zero | Y | Y | Y |
| **Sensing Mode** | Dynamic force | Dynamic force | Static force | Static force | Static force |
| **Sensitivity** | 16 mV⋅kPa^-1^ | 0.63 mV⋅ kPa^-1^ | 8.2 × 10^−5^ ΔR/R(%)⋅ kPa^−1^ | effective gauge factor GF = 0.23 | d(logP)/d(logR) = 2.5 |
| **Sensing Range** | 0-80 kPa | 0-60 kPa | 0-30 kPa | 0-30 kPa | 0.1-10 kPa |
| **Unit No.** | 3 | 1 | 8 × 8 | 17 | 4 × 36 |
| **Electrode Design Principle** | Sandwich structure | Sandwich structure | Multi-layer | Multi-layer | Multi-layer |
| **Multiplexing Method** | Radial symmetry | N/A | Grid array | Grid array | Helical array |
| **Sensing Coverage** | **Omnidirectional** | **Single direction** | **Inflated ballon** | **Inflated ballon** | **Omnidirectional** |
| **Integration Object** | **Catheter shaft** | **Catheter shaft** | **Ballon** | **Ballon** | **Catheter shaft** |
| **Integration Strategy** | Direct integration onto a custom-designed catheter shaft | Patch-type sensor mounted on the catheter surface with external wiring | Sensor attached to the balloon surface with external wiring | Sensor attached to the balloon surface with external wiring | Patch-type sensor mounted onto the catheter surface with external wiring |
| **Dimension** | For 2.8 mm OD. Catheter (customizable) | For 3.33 mm OD catheter | 10 mm × 10 mm | ~16 mm× ~14 mm | For 0.6 mm OD catheter |
| **Applications** | All catheter-based interventions | All catheter-based interventions | Electrode ballon catheter | Electrode ballon catheters | Thin medical catheters |

**Literature review on the typical human lumens in various endoluminal interventions**

**Table S2. Typical sizes of human lumens in different endoluminal interventions**

| **Anatomy** | **Mean Transverse Diameter**  **(Male)** | **Mean Transverse Diameter**  **(Female)** | **Ref.** |
| --- | --- | --- | --- |
| *Cardiovascular Interventions* | | | |
| Abdominal aorta (i.e., descending aorta, ascending aorta, etc.) | 18.7±2.7 mm - 34.1±3.9 mm | 16.0±1.7 mm -31.9±3.5 mm | ^7^ |
| Coronary arteries | 2.2±0.5 mm -4.5±0.5 mm | 1.8±0.5 mm – 3.9±0.4 mm | ^8^ |
| Inferior vena cava | 19±5 mm | 17±5mm | ^9^ |
| *Respiratory Interventions* | | | |
| Main Bronchi | 13.4±2.3 mm | 11.6±2.0 mm | ^10^ |
| Trachea | 17.1±3.6 mm | 15.7±2.1 mm | ^10^ |
| *Gastrointestinal Interventions* | | | |
| Small intestine | 16.4±2.8 mm – 19.0±2.3 mm | | ^11^ |
| Colon | 3.5±0.8 mm – 8.1±2.1 mm | 3.6±0.5 mm – 9.6±1.8 mm | ^12^ |

**Literature review on the typical interaction pressure in various endoluminal interventions**

**Table S3. Typical pressure or force range during tissue-catheter interactions in various endoluminal interventions**

| **Category** | **Anatomy / Motion Source** | **Reported Pressure or Force Range** | **Relevance to This Study** | **Ref.** |
| --- | --- | --- | --- | --- |
| Respiratory intervention | Main bronchi / trachea | Peak airway pressure: ~1.5–3.9 kPa  Frequency: 12-20 breaths per minute | Context for bronchial phantom narrowing and protrusion contact | ^13^ |
| Physiological motion | Blood pressure / arteries | Diastolic blood pressure: ~80 mmHg  (10.7 kPa)  Systolic blood pressure: ~120 mmHg  (16.0 kPa)  In healthy adults. | Dynamic background in vascular intervention settings | ^14^ |
| Physiological motion | Vascular pulsation / arteries | 40 mmHg (5.3 kPa)  Frequency: 60-120 beats per minute | Dynamic background in vascular intervention settings | ^14^ |
| Physiological motion | Blood pressure / veins | 8-12 mmHg  (0.3-1.6 kPa) | Dynamic background in vascular intervention settings | ^15^ |
| Cardiovascular intervention | Vessels | Contact force:  ~0.10–1.59 N | Context for localized catheter–wall contact in the phantom study | ^16,17^ |
| Cardiovascular intervention | Vessels | Contact pressure:  <100 kPa | Reference for catheter–vessel wall interaction | ^18^ |

**Reference**

1	Bargain, F., Panine, P., Santos, F. D. D. & Tencé-Girault, S. From solvent-cast to annealed and poled poly(VDF-co-TrFE) films: New insights on the defective ferroelectric phase. *Polymer* **105**, doi:10.1016/j.polymer.2016.10.010 (2016).

2	Yokoyama, K. *et al.* Novel Contact Force Sensor Incorporated in Irrigated Radiofrequency Ablation Catheter Predicts Lesion Size and Incidence of Steam Pop and Thrombus. *Circulation: Arrhythmia and Electrophysiology*, doi:10.1161/CIRCEP.108.803650 (2008).

3	Gil, B., Li, B., Gao, A. & Yang, G.-Z. Miniaturized Piezo Force Sensor for a Medical Catheter and Implantable Device. *ACS Applied Electronic Materials* **2**, 2669–2677, doi:10.1021/acsaelm.0c00538 (2020).

4	Han, M. *et al.* Catheter-integrated soft multilayer electronic arrays for multiplexed sensing and actuation during cardiac surgery. *Nature Biomedical Engineering 2020 4:10* **4**, doi:10.1038/s41551-020-00604-w (2020).

5	Kim, D.-H. *et al.* Electronic sensor and actuator webs for large-area complex geometry cardiac mapping and therapy. *Proceedings of the National Academy of Sciences* **109**, doi:10.1073/pnas.1205923109 (2012).

6	Sekitani, T. *et al.* Flexible organic transistors and circuits with extreme bending stability. *Nature Materials 2010 9:12* **9**, doi:10.1038/nmat2896 (2010).

7	Rogers, I. S. *et al.* Distribution, determinants, and normal reference values of thoracic and abdominal aortic diameters by computed tomography (from the Framingham Heart Study). *Am J Cardiol* **111**, 1510–1516, doi:10.1016/j.amjcard.2013.01.306 (2013).

8	Dodge, J. T., Jr., Brown, B. G., Bolson, E. L. & Dodge, H. T. Lumen diameter of normal human coronary arteries. Influence of age, sex, anatomic variation, and left ventricular hypertrophy or dilation. *Circulation* **86**, 232–246, doi:10.1161/01.cir.86.1.232 (1992).

9	Taniguchi, T. *et al.* Impact of Body Size on Inferior Vena Cava Parameters for Estimating Right Atrial Pressure: A Need for Standardization? *J Am Soc Echocardiogr* **28**, 1420–1427, doi:10.1016/j.echo.2015.07.008 (2015).

10	Olivier, P., Hayon-Sonsino, D., Convard, J. P., Laloe, P. A. & Fischler, M. Measurement of left mainstem bronchus using multiplane CT reconstructions and relationship between patient characteristics or tracheal diameters and left bronchial diameters. *Chest* **130**, 101–107, doi:10.1378/chest.130.1.101 (2006).

11	Pallotta, N., Baccini, F. & Corazziari, E. Contrast ultrasonography of the normal small bowel. *Ultrasound Med Biol* **25**, 1335–1340, doi:10.1016/s0301-5629(99)00103-9 (1999).

12	Bhatnagar, B. N., Sharma, C. L., Gupta, S. N., Mathur, M. M. & Reddy, D. C. Study on the anatomical dimensions of the human sigmoid colon. *Clin Anat* **17**, 236–243, doi:10.1002/ca.10204 (2004).

13	Lemyze, M. *et al.* Ventilator settings for fiberoptic bronchoscopy during mechanical ventilation: a randomized adjudicator-blinded controlled trial VentSetFib. *Critical Care* **30**, doi:10.1186/s13054-026-05847-8 (2026).

14	Tang, K. S., Medeiros, E. D. & Shah, A. D. Wide pulse pressure: A clinical review. *The Journal of Clinical Hypertension* **22**, 1960–1967, doi:10.1111/jch.14051 (2020).

15	Shah, P. & Louis, M. A. in *StatPearls*	(2026).

16	Chi, W. *et al.* Learning-based endovascular navigation through the use of non-rigid registration for collaborative robotic catheterization. *International Journal of Computer Assisted Radiology and Surgery* **13**, 855–864, doi:10.1007/s11548-018-1743-5 (2018).

17	Rafii-Tari, H. *et al.* Reducing contact forces in the arch and supra-aortic vessels using the Magellan robot. *Journal of Vascular Surgery* **64**, 1422–1432, doi:10.1016/j.jvs.2015.06.215 (2016).

18	Moriwaki, T. *et al.* In Vitro Measurement of Contact Pressure Applied to a Model Vessel Wall during Balloon Dilation by Using a Film-Type Sensor. *J Neuroendovasc Ther* **16**, 192–197, doi:10.5797/jnet.oa.2021-0068 (2022).
